# Cost-Effectiveness Models of Proton Therapy for Head and Neck: Evaluating Quality and Methods to Date

**DOI:** 10.14338/IJPT-20-00058.1

**Published:** 2021-06-25

**Authors:** Danmeng Huang, Steven J. Frank, Vivek Verma, Nikhil G. Thaker, Eric D. Brooks, Matthew B. Palmer, Ross F. Harrison, Ashish A. Deshmukh, Matthew S. Ning

**Affiliations:** 1 Department of Radiation Oncology, University of Texas MD Anderson Cancer Center, Houston, TX, USA; 2 Department of Management, Policy and Community Health, School of Public Health, University of Texas Health Science Center, Houston, TX, USA; 3 Arizona Oncology, Tucson, AZ, USA; 4 University of Florida Health Proton Therapy Institute, Jacksonville, FL, USA; 5 Legion Healthcare Partners, Houston, TX, USA; 6 Department of Gynecologic Oncology, University of Texas MD Anderson Cancer Center, Houston, TX, USA

**Keywords:** oropharyngeal cancer, comparative analysis, health economics, health care value, proton beam therapy

## Abstract

**Purpose:**

Proton beam therapy (PBT) is associated with less toxicity relative to conventional photon radiotherapy for head-and-neck cancer (HNC). Upfront delivery costs are greater, but PBT can provide superior long-term value by minimizing treatment-related complications. Cost-effectiveness models (CEMs) estimate the relative value of novel technologies (such as PBT) as compared with the established standard of care. However, the uncertainties of CEMs can limit interpretation and applicability. This review serves to (1) assess the methodology and quality of pertinent CEMs in the existing literature, (2) evaluate their suitability for guiding clinical and economic strategies, and (3) discuss areas for improvement among future analyses.

**Materials and Methods:**

PubMed was queried for CEMs specific to PBT for HNC. General characteristics, modeling information, and methodological approaches were extracted for each identified study. Reporting quality was assessed via the Consolidated Health Economic Evaluation Reporting Standards 24-item checklist, whereas methodologic quality was evaluated via the Philips checklist. The Cooper evidence hierarchy scale was employed to analyze parameter inputs referenced within each model.

**Results:**

At the time of study, only 4 formal CEMs specific to PBT for HNC had been published (2005, 2013, 2018, 2020). The parameter inputs among these various Markov cohort models generally referenced older literature, excluding many clinically relevant complications and applying numerous hypothetical assumptions for toxicity states, incorporating inputs from theoretical complication-probability models because of limited availability of direct clinical evidence. Case numbers among study cohorts were low, and the structural design of some models inadequately reflected the natural history of HNC. Furthermore, cost inputs were incomplete and referenced historic figures.

**Conclusion:**

Contemporary CEMs are needed to incorporate modern estimates for toxicity risks and costs associated with PBT delivery, to provide a more accurate estimate of value, and to improve their clinical applicability with respect to PBT for HNC.

## Introduction

Proton beam therapy (PBT) is increasingly used because of superior dosimetric characteristics relative to photon therapy [1–8], such as intensity-modulated radiation therapy (IMRT), resulting in lower doses and decreased complications among healthy tissues [9–13]. This unique advantage is particularly welcome for the treatment of head-and-neck cancers (HNCs), anatomically complex tumors that closely approximate sensitive organs at risk. The prevalence of human papillomavirus (HPV)-associated HNCs continues to rise [14, 15], particularly among young and otherwise healthy patients who live decades after treatment and may, thus, benefit most from PBT. However, the advanced technology necessary for PBT delivery is accompanied with greater cost relative to photons. However, although associated with upfront costs, PBT has the potential to contribute value to cancer care if costs associated with toxicity management are lowered.

In this era of value-based health care, formal economic evaluations are warranted to address such questions, to inform optimal management, and to guide technologic investments. Cost-effectiveness models (CEMs) portray the transition from diagnosis through treatment and then toward various outcomes and/or toxicity states (eg, stable disease, local or distant failure, death, and acute or late toxicities) [16]. The most-common CEM is the Markov decision-analytic model, starting with a hypothetical population with prespecified disease, prognosis, and treatment parameters. After their assigned intervention (eg, PBT versus IMRT), patients will probabilistically transition through various states during posttreatment follow-up.

Survival across their natural history is evaluated for quantity (duration) and quality: each health state is assigned a quantitative utility value (a fraction of 1, which is associated with the “optimal” state: absence of death [“0”], disease, or morbidity). These utilities are then multiplied by the duration lived within each respective state to calculate quality-adjusted life years (QALYs)—for example, 1 year of dysphagia × 0.7 (the utility value assigned to dysphagia) = 0.7 QALYs (or 0.3 QALYs lost by experiencing that state). These QALYs are aggregated across each cohort arm at prespecified time points (eg, 1, 3, or 5 years) and are compared to determine the relative QALYs gained (or lost) by each intervention.

At the same time, medical costs associated with initial treatment, toxicity management, and subsequent therapy are aggregated across each cohort arm (at the same prespecified time points) and are compared to determine the differential cost associated with each intervention. Final comparison of their value requires calculation of the incremental cost-effectiveness ratio (ICER), which represents the relative cost of each intervention compared with the relative QALYs gained. Note that although an intervention may not appear cost-effective during the early phase (eg, because of equivalent acute toxicities), it can demonstrate greater value over time via sustained benefits in disease-related or late-toxicity outcomes (if survival is adequately maintained).

However, uncertainties in the assumptions underlying the CEMs can limit interpretation and real-world applicability. Given their increasing literature presence and consideration among modern health care evaluations, the objectives of this review are to (1) assess the reporting and methodological quality of existing cost-effectiveness studies on PBT for HNC, (2) critique the parameter inputs referenced within those models, (3) evaluate the suitability of those models for guiding clinical and economic strategies, and (4) summarize areas for improvement and future direction among successive CEMs.

## Materials and Methods

PubMed (National Center for Biotechnology Information, Bethesda, Maryland) was queried for CEMs specific to PBT for HNC. Search terms were restricted from 2000 through 2020 and included the following headings: head-and-neck, oropharynx, thyroid, salivary, larynx, hypopharynx, oral cavity, nasal, orbit, nasopharynx, paranasal, protons, proton radiation, proton beam, cost, cost-effectiveness, value, and economics. Four full CEMs specific to HNC were identified [17–20], which (1) compared PBT to any comparators, and (2) expressed results in terms of cost per QALY gained. The bibliography of each CEM was reviewed, and subsequent citations were tracked in Scopus (Elsevier, Amsterdam, the Netherlands) to identify any newly published studies meeting inclusion criteria. General characteristics, modeling information, and methodological approaches were extracted for each identified study [17–20].

### Reporting Quality

The Consolidated Health Economic Evaluation Reporting Standards (CHEERS) checklist [21] was developed through a modified-Delphi technique by the International Society for Pharmacoeconomics and Outcomes Research (Lawrenceville, New Jersey) Health Economic Evaluation Publication Guidelines Good Reporting Practices Task Force. It has 24 items assessing the consistency and transparency of reporting economic evaluations, aiming to achieve standardized reporting of economic evaluation.

### Methodological Quality

The Philips checklist [22] was selected as the tool to measure methodological quality because it was specifically designed for the assessment of modeling studies and is recommended by both the National Institute for Health and Care Excellence (NICE; London, England) [23] and the Cochrane Collaboration (London, England) [24]. Note that this checklist was not intended to comprehensively standardize the decision model–development process, functioning more as a series of consolidated good practice statements to consider in appraising decision models.

### Assessment of CEM Parameters

A hierarchy scale developed by Cooper et al [25] was used to assess the quality of parameter inputs from data sources referenced in the CEM studies. The scale ranks the quality of evidence from 1 (best quality) to 6 (lowest quality) for studies of clinical effect and safety, medical resource use, health care costs, and utility scores.

## Results

### Summary of CEM Studies (Tables 1–5)

#### Lundkvist et al [26]

The Swedish Markov cohort model simulated several histologies including 300 patients with HNC comparing PBT and conventional radiotherapy (CBT) from a societal perspective. This simplified disease model had only 3 health states: healthy, chronic adverse event conditions, and death. Acute mucositis and xerostomia, as well as chronic xerostomia, were considered but only for their contribution to mortality risk. In conclusion, PBT was simulated to contribute an additional 1.02 QALYs at the cost of 3 887 (US $3 693), which translated to 3 800 (US $3 693) per QALY in 2002. The authors concluded that PBT was cost effective, assuming a willingness-to-pay level of 55 000 (US $52 250).

**Table 1.** Summary of cost-effectiveness modeling studies (model structure).

**Source, y**	**Country**	**Cancer type**	**Model structure**						
			**Comparators**	**Perspective**	**Health states**	**Side effects**	**Sensitivity analysis**	**Handle heterogeneity**	**Assumptions**
Lundkvist et al, 2005 [26]	Sweden	HNCs such as cancer in the hypopharynx	1. PBT	Societal	1. Healthy	1. Acute mucositis	Scenario analyses (One-way sensitivity analyses)	None	1. Patients assumed mortality of normal population if they survived >9 y
			2. CRT		2. Chronic adverse event conditions	2. Acute xerostomia			2. No gains in quality of life for PBT
					3. Death	3. Chronic xerostomia			
Ramaekers et al, 2013 [27]	Netherlands	Locally advanced (stage III-IV) HNC with grade <2 dysphagia and xerostomia	1. IMPT for all patient	Dutch health care	1. Disease free with no toxicity	1. Xerostomia	1. Scenario analysis: relax the assumption of equal disease progression for IMPT and IMRT	For IMPT, if efficient strategy, individual cost effectiveness was first calculated to determine IMPT/IMRT	1. Disease progression and survival were assumed equal for IMPT and IMRT based on clinical evidence
			2. IMRT for all patients		2. Disease free with grade ≥2 toxicity (1 health state for each side effect, 3 in total)	2. Dysphagia	2. Probabilistic sensitivity analyses		2. Acute toxicity is reversible during the first 6 mo after radiation therapy; chronic toxicity is irreversible
			3. IMPT if efficient		3. Locoregional recurrence	3. Dysphagia and xerostomia	3. EVPI analysis		3. There was no transition from locoregional recurrence to death via distant metastasis because the mortality rate of locoregional recurrence already accounted for <1 y survival with distant metastasis
					4. Distant metastasis				
Sher et al, 2018 [28]	United States	Oropharyngeal SCC	1. PBT	Payer (Medicare) and societal	1. No evidence of disease with PEG	1. Xerostomia	1. One-way sensitivity analysis	Populations by HPV status	1. The oncologic outcomes were assumed to be identical between IMRT and PBT
			2. IMRT		2. No evidence of disease without PEG	2. Dysphagia	2. Probabilistic sensitivity analysis		2. Similar late recurrence or toxicity risk, except for potential improvements in xerostomia, dysphagia, and dentition, as detailed, were assumed between IMRT and PBT
					3. Locoregional recurrence with salvage	3. Dentition	3. EVPI analysis		
					4. Locoregional recurrence without salvage	4. PEG			
					5. Distant metastasis				
					6. Dead (other)				
					7. Dead (OPC)				
Li et al, 2020 [20]	China	Paranasal sinus and nasal cavity cancers	1. IMPT	Chinese health care	1. No cancer	None	1. One-way sensitivity analysis	Stratified analysis by age	Other than efficacy in eradicating cancer, clinical outcomes including irradiation-induced acute and late toxicities, were assumed the same between IMPT and IMRT
			2. IMRT		2. Alive with cancer (include recurrence, metastasis, or residue)		2. Probabilistic sensitivity analysis		2. Transition probability from “no cancer” to “alive with cancer” was assume to vary over time
					3. Death		3. Stratified analyses		
							4. Tested willingness-to-pay at $30 828, $50 000, and $100 000		

**Abbreviations:** HNC, head and neck cancer; PBT, proton beam therapy; CRT, conventional radiotherapy; IMPT, intensity-modulated proton therapy; IMRT, intensity-modulated radiation therapy; EVPI, expected value of perfect information; SCC, squamous cell carcinoma; PEG, percutaneous endoscopic gastrostomy; HPV, human papillomavirus; OPC, oligodendrocyte progenitor cell.

**Table 2.** Summary of cost-effectiveness modeling studies (costs).

**Source, y**	**Country**	**Model inputs**				
		**Currency, y**	**Cost values**	**Cost type**	**Notes**	**Cost sources**
Lundkvist et al, 2005 [26]	Sweden	Euro, 2002	1. Total radiation cost: PBT 13 049 ($12 396.55 converted) CRT 5 477 ($5 203.15 converted)	1. Investment cost for a proton facility	Annuitized one-time investment to a yearly cost assuming lifetime of 30 y and interest rate at 5%	Literature
				2. Operation cost	For PBT and CRT	Literature
				3. Transportation and hotel accommodation	Assumed for 35% of PBT patients	
			2. Dental cost: first y, 1 608.7 ($1 528.625 converted); then, 271.7/y ($258.115 converted)	4. Cost saving from reduced dental visits	Assumption and literature-based	
Ramaekers et al, 2013 [27]	Netherlands	Euro, 2010		1. Treatment costs for IMPT	Multiplied IMRT treatment costs with a cost ratio of 2.1	Published cost analyses
				2. Toxicity independent/dependent resource use and unit cost	Activity-based costing	Guidelines, list of tariffs, expert opinion
Sher et al, 2018 [28]	United States	USD, 2016		1. Treatment costs	For both, payer perspective only; activity-based costing: CPT codes	Medicare, physician fee, schedule and outpatient prospective payment system
				2. Cost of building and financing proton facility	For societal perspective only	Administrative claims data analysis
				3. Monthly costs after recurrence		
Li et al, 2020 [20]	China	USD, 2020	$50 000	1. Treatment cost for IMPT	Estimated based on 32 fractions to a total dose of 70 Gy.	Estimate provided by a proton center
			$12 000	2. Treatment cost for IMRT		Estimate provided by a cancer center
			$5 000	3. Treatment cost for concurrent chemotherapy	Estimated based on 3 cycles of 80–100 mg/m^2^ cisplatin bolus injection delivered on d 1, 21, and 42 of the radiotherapy	Estimate provided by a cancer center
			$1 000	4. Follow-up cost/y	Included hematologic and biochemistry profiles, nasopharyngeal fiberoptic endoscope examination, MRI of head and neck, chest radiography, and abdominal ultrasonography	Estimate provided by a cancer center
			$5 000	5. Cost for palliative therapy/y	Estimated based on 8 cycles of oral palliative chemotherapy with 5-fluorouracil	Estimate provided by a cancer center

**Abbreviations:** PBT, proton beam therapy; CRT, conventional radiotherapy; CPT, current procedural terminology; USD, United States dollar; IMPT, intensity-modulated proton therapy; IMRT, intensity-modulated radiation therapy; MRI, magnetic resonance imagining.

**Table 3.** Summary of cost-effectiveness modeling studies (utilities).

**Source, y**	**Country**	**Model Inputs**		
		**Utility values**	**Utility category**	**Utility sources**
Lundkvist et al, 2005 [26]	Sweden	0.75	Utility score of patients with HNC 2–3 y after diagnosis	Literature
Ramaekers et al, 2013 [27]	Netherlands		EQ-5D in Dutch patients with HNC	Cross-sectional survey
Sher et al, 2018 [28]	United States		1. Utilities for health states	Cross-sectional study on healthy subjects using the standard gamble method
			2. Disutilities for toxicities	Cross-sectional study on in patients with HNC using EQ-5D
Li et al, 2020 [20]	China	0.94	1. Utility score without cancer	Cross-sectional survey on patients with SCC of the upper aerodigestive tract in remission using Time Trade-Off method
		0.47	2. Utility score with cancer	

**Abbreviations:** HNC, head and neck cancer; EQ-5D, EuroQol 5-Dimension; SCC, squamous cell carcinoma.

**Table 4.** Summary of cost-effectiveness modeling studies (probabilities).

**Source, y**	**Country**	**Model inputs**		
		**Probability category**	**Probability notes**	**Probability sources**
Lundkvist et al, 2005 [26]	Sweden	1. Overall mortality		Literature
		2. RR mortality: 0.76	Assumed based on studies of hyperfractionation	Literature
Ramaekers et al, 2013 [27]	Netherlands	1. Toxicity probability of xerostomia or dysphagia	The required dose parameters used to calculate probabilities of toxicity were identified from a planning study of 25 patients. Averaged probabilities were applied to each strategy	Probabilities of toxicity estimated based on 2 published NTCP models
		2. Disease progression		Meta-analysis
		3. Mortality from locoregional recurrence		RTOG 9610
		4. Mortality from distant metastases		Prospective study
Sher et al, 2018 [28]	United States	1. RR of dysgeusia: 0.75	PBT vs. IMRT in y 1	Assumption
		2. RR of PEG dependence: 0.75	PBT vs. IMRT in y 1	
		3. RR of xerostomia: 0.75	PBT vs. IMRT after y 1	
		4. Additional risk of dental complications	IMRT vs. PBT at 1 y	Literature-based assumption
		5. Progression	1. NED to LRR by HPV status	RTOG 0129 trial
			2. NED to DM	RTOG 0129 trial
		6. Mortality rates	LRR, DM	Retrospective analysis of RTOG 0129 and RTOG 0522
Li et al, 2020 [20]	China	1. Probabilities eradicating cancer	Different for IMPT and IMRT	Systematic literature reviews
		2. Disease progression	“No cancer” to “alive with cancer”	
		3. Cancer mortality	“Alive with cancer” to “death”	
		4. Noncancer mortality		2016 Life Tables of United States

**Abbreviations:** RR, relative risk; NTCP, normal tissue complication probability; RTOG, Radiation Therapy Oncology Group; PBT, proton beam therapy; IMRT, intensity-modulated radiation therapy; PEG, percutaneous endoscopic gastrostomy; NED, no evidence of disease; LRR, locoregional recurrence; HPV, human papillomavirus; DM, distant metastasis; IMPT, intensity-modulated proton therapy.

**Table 5.** Summary of cost-effectiveness modeling studies (results).

**Source, y**	**Country**	**Model results**				
		**Δ Cost**	**Δ QALYs**	**ICER**	**Notes**	**Sensitivity analysis**
Lundkvist et al, 2005 [26]	Sweden	3 887 ($3 692.65 converted)	1.02	3 800 ($3 610 converted)		Results are sensitive to less favorable hazard rate, exclusion of dentistry cost savings, and shorter proton facility lifetime
Ramaekers et al, 2013 [27]	Netherlands	1. 2 612 ($3 473.96 converted); IMPT if efficient versus IMRT	0.043; IMPT if efficient versus IMRT	60 278 ($80 169.74 converted)		1. In alternative scenario, IMRT for all patients yielded more QALYs and was less expensive and thus was the dominant strategy
		2. 7 339 ($9 760.87 converted); IMPT for all versus IMPT if efficient	0.057 IMPT for all versus IMPT if efficient	127 946 ($170 168.2 converted)		2. The value of further research emphasizes utility scores after xerostomia, NTCP models for dysphagia and for xerostomia
Sher et al, 2018 [28]	United States	1. $20 164	0.07	$288 000/QALY	Payer perspective, HPV^+^ patients	1. In the HPV+ population, the ICER fell below $100 000/QALY if proton therapy reduced risk of xerostomia by 84%; proton therapy was not cost effective for patients <55 y, except both xerostomia- and PEG-dependence risks were halved
		2. $27 311		$390 000/QALY	Societal perspective, HPV^−^ patients	2. In the HPV^−^ population, the ICER was always above $100 000/QALY, even in unrealistic conditions that strongly favored proton therapy
		1. $20 640	0.04	$516 000	Payer perspective, HPV^+^ patients	3. Resolving the specific parameters of proton versus photon radiation therapy will add no additional value to the comparison, which favors IMRT at baseline at a societal WTP of $100 000/QALY
		2. $27 787		$695 000/QALY	societal perspective, HPV-negative patients	
Li et al, 2020 [20]	China	$38 928.7	1.65	$23 611.2/QALY		1. One-way sensitivity analysis showed top 3 influential parameters that may change the cost effectiveness of IMPT: the probability of IMPT eradicating cancer, the probability of IMRT eradicating cancer, and the cost of IMPT
						2. IMPT was cost effective in patients 56 y and younger at the base case WTP of China

**Abbreviations:** QALY, quality-adjusted life years; ICER, incremental cost-effectiveness ratio; IMPT, intensity-modulated proton therapy; IMRT, intensity-modulated radiation therapy; NTCP, normal tissue complication probability; HPV, human papillomavirus; PEG, percutaneous endoscopic gastrostomy; WTP, willingness-to-pay.

#### Ramaekers et al [27]

The Dutch Markov cohort model simulated 25 patients with stage III–IV HNCs (oral cavity, laryngeal, and pharyngeal tumors) from the Dutch health care perspective, comparing intensity-modulated PBT (IMPT) with IMRT. This model better reflected the natural history of cancer by including the following health states: disease free without toxicity, disease free with toxicity, locoregional recurrence, distant progression, and death. However, toxicities focused solely on grade 2 or greater dysphagia and xerostomia, with parameter inputs based on normal tissue complication probability (NTCP) models and comparative plan studies, not empiric clinical data.

In addition to evaluating IMPT for the whole study population, a subset cohort (PBT “if efficient”) was analyzed in which patients were stratified to the most cost-effective modality under a willingness-to-pay threshold of 80 000 (US $106 400) in 2010. Employing this case-by-case strategy, the authors concluded that PBT could be cost effective but yielded a mere 0.043 QALYs gained at the additional cost of 2 612 (US $3 474) (translating to an ICER of 60 278 [US $80 170] per QALY). However, on sensitivity analysis, which differentiated disease and survival outcomes among PBT versus IMRT, the latter dominated with more QALYs at lower cost when considering all patients.

#### Sher et al [28]

The American Markov model compared PBT to IMRT for stage IVA oropharyngeal carcinoma, structuring the model on the natural cancer history of a single HNC patient, upon which various sensitivity analyses were applied. Separate model inputs were applied to simulate HPV^+^ versus HPV^−^ subpopulations, with external calibration of disease-related outcomes against independently published data. Toxicity endpoints (acute dysgeusia, late grade ≥2 xerostomia, percutaneous endoscopic gastrostomy [PEG]-tube placement, and dental complications) once again incorporated hypothetical modeling inputs from retrospective clinical studies. The estimated benefit with PBT assumed a symmetric triangular distribution for reduction for each toxicity endpoint (ranging from 0% to 50%), with a “best-case scenario” defined as a maximal 50% improvement. With significant limitations, the authors concluded that PBT, in general, was not cost effective from either the payer or societal perspectives (at a threshold of US $100 000 in 2016) and would only be cost effective from the payer perspective under favorable conditions for young HPV^+^ patients.

#### Li et al [20]

The Chinese Markov cohort model compared IMPT versus IMRT for treating paranasal sinus and nasal cavity cancers from a Chinese health care perspective. The model adopted a simple structure of only 3 health states: no cancer, alive with cancer (including recurrent, metastatic, or residual disease), and death. Interestingly, no acute or long-term side effects were included because the authors assumed similar toxicity outcomes among IMPT and IMRT; however, the modalities were assumed to yield different disease control outcomes (in favor of IMPT). Evaluation started with the base-case assessment of a 47-year-old patient, along with stratified analyses for different age subgroups, altogether indicating cost effectiveness for patients 56 years or younger (including the base case), at a threshold of $30 828 specific to China.

### Reporting Quality (Supplemental Material Table S1)

The 4 CEMs generally adhered to the CHEERS checklist criteria [21], although each lacked certain components. For example, only the Sher et al [28] study indicated both (1) study comparators, and (2) evaluation type within their title. The CHEERS checklist also requires inclusion of objectives, perspective, setting, methods, results, and conclusions in the abstract—with at least one component overlooked by each study. Overall, methodology reporting would have been stronger if references were provided justifying the rationale for model design, as well as input selection (such as base case population, time horizon, and discount rate). Specifically, the Lundkvist et al [26] study provided no support for use of the Markov cohort methods, and the Ramaekers et al [27] study failed to represent uncertainties among model distributions. The Sher et al [28] study lacked both criteria and failed to explore the actual generalizability of their findings (or lack thereof). The Li et al [20] study did not report mean estimated outcomes or costs for each radiation therapy and lacked recognition of and discussion of previous studies [26, 27]

### Methodological Quality (Supplemental Material Table S2)

In terms of structural design, each CEM simplified their models with numerous assumptions to the extent of inadequately reflecting the comprehensive natural history of HNC. Across all 4 CEMs, the duration of treatment effect was left ambiguous—a major limitation given the significant effect on calculated QALYs, ICERs, and conclusions—with varying cycle times applied to each model, for which only the Ramaekers et al [27] study provided justification (and extrapolated the short-term effect as an alternative assumption tested on sensitivity analysis). The Sher et al [28] constructed their model based off the history of a 65-year-old patient with HNC, on top of which various sensitivity models were applied. Three CEMs, except for Lundkvist et al [26] provided justification for model structure, although the Ramaekers et al [27] model provided no reference, and the Li et al [20] model was based only on an older published study. All 4 CEMs directed appropriate attention toward significant parameters; however, exact methods of data identification, selection, and quality assessment were unreported. In the setting of multiple data sources, no systematic approaches were applied toward evidence synthesis, and all CEMs appeared to lack internal consistency checks.

With respect to cost inputs, the Lundkvist et al [26] and Sher et al [28] studies presented evaluations from the societal perspective, but focused mainly on the cost of building and/or financing a proton facility and omitting other essential elements for comprehensive analysis. Per the Second Panel on Cost-Effectiveness in Health and Medicine [29], the societal perspective should (1) cover all parties affected, and (2) include all significant costs incurred (direct and indirect). Direct cost estimates among these studies failed to consider out-of-pocket patient expenses, for example, and only the Lundkvist et al [26] study considered direct nonmedical costs (eg, transportation, accommodations, among others) associated with acquisition of provider services. The most important nonmedical cost—lost productivity—was poorly captured by existing CEMs as well. Finally, none of the CEMs clearly specified the primary decision maker for analysis.

### CEM Parameter Assessment (Supplemental Material Table S3)

Dating back to 2005, the Lundkvist et al [26] study generated the simplest model with the literature sources available at the time. Survival rates were estimated from the Swedish cancer registry, with the relative mortality risk of PBT based purely off assumptions. The Li et al [20] study, from 2020, assumed differential probabilities eradicating disease in the setting of paranasal sinus and nasal cavity cancers [30]. In contrast, both the Ramaekers et al [27] and Sher et al [28] studies (in 2013 and 2018, respectively) generally considered PBT and IMRT to be analogous with respect to disease and/or survival outcomes (although the Ramaekers et al [27] study also explored the alternative scenario in the sensitivity analysis). In Ramaekers et al [27], disease progression probabilities were based on a meta-analysis of randomized controlled trials examining arms comprising radiation with concomitant chemotherapy. Cancer-related mortalities from locoregional recurrence and distant metastases were from a single randomized controlled trial (Radiation Therapy Oncology Group [RTOG], Philadelphia, Pennsylvania; RTOG 9610) and a prospective study, respectively. The Sher et al [28] study used more-recent data of RTOG 0129 and RTOG 0522 for disease progression and mortality rates, whereas the Li et al [20] study incorporated probabilities and rates from systematic review and meta-analysis data [30].

With respect to complications, the Lundkvist et al [26] study completely excluded toxicity considerations for HNC. Similarly, the Li et al [20] study assumed no differential outcomes in irradiation-induced acute and late toxicities between IMPT and IMRT for paranasal sinus and nasal cavity cancers. Both the Ramaekers et al [27] and Sher et al [28] studies attempted to apply clinically relevant data from NTCP modeling studies, but again, these theoretical models estimate adverse-event probabilities based on dosimetric plan comparisons and are beset by their own uncertainties in the absence of clinical validation. The Sher et al [28] study did also incorporate a couple of small retrospective series available at the time, but still entailed significant, unsupported assumptions for relative toxicity risks.

Further issues include the poor quality of utility or disutility values across each of these studies. Although the utility value in the Lundkvist et al [26] study was referenced to several studies, the actual value associated with them was not readily traceable. The Ramaekers et al [27] study incorporated a very indirect measure of utilities from EuroQol 5-Dimension (EQ-5D; EuroQol Research Foundation, Rotterdam, the Netherlands) data among HNC patients, whereas the Sher et al [28] study provided direct measurements derived from healthy subjects via the standard gamble method. Utility values in Li et al [20] stemmed from cross-sectional data.

Finally, regarding costs, medical resource use across models was mainly based on expert opinion or quantities reported among procedural guidelines, in lieu of direct observation, whereas preexisting literature sources were cited for radiotherapy treatment and cancer-related costs. Two studies employed activity-based costing (Ramaekers et al [27] and Sher et al [28]), citing government-issued fee schedules as data sources for unit costs. Both Lundkvist et al [26] and Sher et al [28] also evaluated the societal perspective, estimating the investment cost for a proton therapy center: calculations in the former were based off very outdated literature [26], whereas the latter [28] failed to specify relevant sources (raising questions of validity). Of all the studies, only Lundkvist et al [26] included patient-level transportation and hotel accommodation costs (although even those figures were based on assumptions). Cost estimates from a proton center and cancer center in China were used in Li et al [20].

## Discussion

In this modern era of value-based health care, formal economic evaluations can be useful tools to compare novel interventions and to provide preliminary rationale for management decisions [16]. However, as demonstrated in this review, CEMs are not without their limitations. These hypothetical simulations are largely bound by the quality of available evidence for reference as inputs. Although quantitative in nature, subjective choices in design, methodology, and parameters can drastically affect the quality and applicability of findings.

These comments aside, the referenced authors are to be applauded for their innovative efforts during the early era of PBT, when few, if any, high-quality studies existed in the literature. In general, CEMs are notoriously difficult to conduct because of their comprehensive nature and difficulties with simultaneously accounting for each outcome and variable of significance. The purpose of this review is to evaluate the methodology of existing models and to identify future directions for improvement. In its simplest form, the health care value equation is defined as quality (or outcomes) over cost, and our suggestions involve both components.

### CEM Development: General Best Practices (Table 6)

Future CEMs should follow the recommendations of the Second Panel on Cost-Effectiveness in Health and Medicine [29], which suggests inclusion of both a health care sector and a societal perspective. For either perspective, all economic and clinical effects of interventions in the impact inventory should be taken into consideration to ensure comprehensive coverage of all consequences for each party. To ensure transparency, all model parameters, assumptions, analyses, and structural decisions should be clearly outlined and supported with appropriate rationale. For critical model inputs that will significantly influence results or model validity, parameter selection should be informed via formal evidence synthesis. Finally, the panel recommends that identical discount rates for both costs and health consequences be explored within sensitivity analyses, in contrast to the heterogeneous rates applied by Ramaekers et al [27].

**Table 6.** Recommended best practices.

**Characteristics**	**Recommended Best Practices**
General	Outline and justify model parameters, assumptions, analyses, and structural decisions
Model structure	Include both health care and societal perspectives
Model inputs	Consider all economic and clinical effects of interventions from the impact inventory
	Use formal evidence synthesis to inform critical model inputs
	Use identical discount rates for both costs and outcomes

### PBT Outcomes: Focus on Toxicity Parameters

Disease-specific outcomes, such as distant metastases and progression-free survival, are presumably similar for PBT and IMRT across multiple disease sites and histologies; thus, no significant concerns were observed for such outcomes. However, the referenced models inadequately represented the most important difference (and major benefit) of PBT over IMRT: decreased toxicities. Lundkvist et al [26] failed to incorporate any toxicity data for HNC because of the paucity of clinical literature at the time, whereas Li et al [20] assumed similar complication rates for paranasal sinus and nasal cavity cancers (despite data indicating decreased toxicities with PBT over IMRT [31, 32]). The other two Markov models were flawed in their choice of toxicity states (with the exclusion of several clinically relevant complications) and suboptimal quality of input values for their included endpoints.

The Ramaekers et al [27] study, similarly because of the lack of data, based parameter inputs off comparative planning studies and NTCP models, which attempt to predict the incidence of treatment-related complications [33]. Admittedly, this was an innovative approach to compare outcomes in the absence of patient-level data [34]; however, those extrapolations of radiobiologic theory are beset by numerous assumptions, which culminate in significant uncertainties and concerns of external validity. Furthermore, only dysphagia and xerostomia were included as toxicity states, with the notable omission of PEG-tube placement (a common and clinically significant treatment-related complication). The NTCP models also underestimated the benefit of PBT versus IMRT with respect to dysphagia (as 18% versus 23%), in contrast to published clinical studies. Follow-up was also quite limited at 1 year, underestimating the long-term value of PBT.

Sher et al [28] improved upon prior models by incorporating additional toxicity endpoints (late xerostomia, acute dysgeusia, PEG-tube placement, and dental complications) and extending the follow-up period for calculation. However, their parameter inputs were again informed by modeling studies and the limited number of small, retrospective series available at the time of study. The benefit of PBT in PEG-tube placement was underestimated as a mere 25% reduction versus IMRT (odds ratio, 0.75), in contrast to published clinical data on patients with oropharyngeal and nasopharyngeal cancer [10,11] (although, admittedly, this endpoint is dependent upon clinician preference and the relative volume of oropharyngeal mucosa in the field). Furthermore, both the Sher et al [28] and the Dutch model [27] focused on grade 2 or greater toxicities, implying moderate symptoms that only modestly interfere with function and may not require intervention. In contrast, grade 3 or greater toxicities (severe symptoms that interfere with daily activity and merit intervention) would arguably be a superior endpoint given their larger impact on both quality and cost. Although some late grade-2 toxicities are clinically significant, appropriate follow-up periods are necessary after treatment to adequately capture them.

At no fault of the authors, pertinent clinical data were quite limited during their period of publication (2005–18), but since then, several robust studies have been published with patient-level data that must be incorporated in future CEMs. For example, a prospective, comparative analysis of IMPT versus IMRT for oropharyngeal cancer demonstrated several benefits of PBT in acute toxicities and patient-reported outcomes (PROs) [10]: notably, significant reductions in PEG-tube use (20% versus 46%), posttreatment hospitalizations (9% versus 31%), and narcotic requirements. Decreased cough, dysgeusia, and dysphagia were also documented on PROs, whereas providers observed less pain and mucositis with IMPT. On an even larger scale, a retrospective comparative effectiveness study of 1483 patients (29% HNCs) [13] linked PBT with a two-thirds reduction in grade 3 or greater adverse events associated with hospital admission (11.5% versus 28%; relative risk [RR], 0.31; 95% confidence interval [CI], 0.15–0.66) and found less detriment to performance status as compared with IMRT (RR, 0.51; 95% CI, 0.37–0.71). To the earlier point emphasizing Common Terminology Criteria for Adverse Events severity, a lower (yet still significant) benefit was observed with grade 2 or greater toxicities (RR, 0.78; 95% CI, 0.65–0.93) as well.

As a final note on outcome parameters, both newer studies already demonstrate the oversight of prior CEMs for several clinically significant and costly endpoints, such as unplanned hospital admissions, pain and corresponding narcotic use, cough, and performance status decline. Other considerations include fatigue, osteoradionecrosis, endocrine complications, fibrosis, esophagitis, and oral mucositis. Furthermore, none of the aforementioned CEMs incorporated PRO data, which can supplement Common Terminology Criteria for Adverse Events assessments to more granularly capture quality-of-life differences [10, 12]. Future CEMs should strive to identify and include such endpoints among their model parameters to provide a more comprehensive demonstration of the true benefit of PBT.

### PBT Costs: Toxicity Management, Treatment Expenses, and Indirect Benefits

Health care costs in the United States are difficult to measure because of varying stakeholder perspectives: that of patients, payers, providers, and society—each of which merits evaluation via distinct scenario analyses. Given their often-conflicting interests, those stakeholders place widely varying emphases on different cost contributors toward decision-making. For example, although fixed costs for research and technology development should always be considered [29], the actual cost of building and financing a proton facility may be more relevant to some groups than others (ie, providers and society, but not patients or payers), given that payers do not directly factor those investment costs into their reimbursement policies.

However, for investment and operational expense of RT delivery, technologic advancements have actually led to dramatic reductions in PBT equipment cost over time [35, 36]. Within the past few years, the massive industrial-sized facilities of the past have transitioned toward smaller, compact units that are readily incorporable within existing medical campuses and are associated with significantly decreased capital investment: from $100-$250 million historically, to now, as low as $25-$30 million per center [35, 37, 38]. Accompanied by optimized delivery efficiency [39], these innovations have simultaneously lowered the threshold for operational sustainability [35, 37, 38], resulting in broader provider adoption and patient access. These same technologic adaptations have also been accompanied by improvements in plan robustness [40], with continued benefits to patient outcomes (and treatment quality). Future CEMs should incorporate contemporary cost figures reflecting such changes, which will certainly enhance the value of PBT relative to historic models.

Comprehensive cost estimates also need to consider direct, as well as indirect, contributors: direct costs are associated with the actual medical services delivered to patients by providers, whereas indirect costs result from disability and productivity loss from disease-related (or treatment-related) morbidity. Optimizing toxicity endpoints, as previously mentioned, would enhance direct estimates by more accurately measuring the cost of treatment-related complications. Indirect costs, on the other hand, are poorly reflected by traditional economic evaluations because of difficulties associated with their measurement. However, the expenses incurred from treatment-related disability and productivity loss are significant contributors to financial toxicity [41], with broad societal implications. These measures are also particularly relevant to PBT because lower treatment-related toxicities could help maintain performance status and work productivity among patients with HNC [13] (many of whom are young working-aged adults). Thus, although challenging, future studies should attempt to incorporate such endpoints, starting, perhaps, with basic work-productivity assessments.

On a final note, CEMs should transition from hypothetical cost estimates toward incorporating real-world figures for cost inputs, just as recommended for clinical outcome and toxicity parameters. From the payer perspective, actual reimbursement records present validated cost data that can differ dramatically from model estimates [42, 43]. For example, our proton therapy center collaborated with stakeholders on an insurance-coverage pilot for PBT, attempting to address patient-access barriers associated with insurance prior authorization [42, 44, 45]. That entailed a comprehensive cost-of-care analysis evaluating total medical charges among case-matched PBT versus IMRT patients, demonstrating no significant differences in average medical costs [42]—a surprising result (among a prospective clinical cohort), which diverged significantly from anticipated cost estimates. Similarly, from the provider perspective, time-driven, activity-based methodologies can measure the actual cost of treatment delivery by methodically quantifying resource use associated with a full care cycle; and from the patient perspective, prospective surveys are useful instruments to assess financial toxicity [41]. Incorporating any of these real-world data sources would bolster the external validity of future CEMs.

## Conclusions

In summary, we identified limitations of the existing CEMs and outlined several areas for improvement among future models. Given their increasing relevance in the modern health care era, economic evaluations should strive to incorporate the highest-quality evidence for parameter inputs (particularly with phase II/III randomized trial data highly anticipated [9, 46]). Collectively, these suggestions will help minimize the uncertainties associated with CEMs, thus providing a more valid and applicable picture of the true value of PBT.
