# Supplementary material for: Cost-Effectiveness Models of Proton Therapy for Head and Neck: Evaluating Quality and Methods to Date
Source: Int J Part Ther. 2021 Jun 25;8(1):339–53. doi: 10.14338/IJPT-20-00058.1 (PMC8270103; doi:10.14338/IJPT-20-00058.1)
Supplement: Supplementary file 1 [file ijpt-08-01-15_s01.docx]

| Supplementary Table 1. CHEERS checklist for reporting economic evaluations of health interventions | | | | | |  | |  |  | |  |
| --- | --- | --- | --- | --- | --- | --- | --- | --- | --- | --- | --- |
| Item No. | Section/item | Recommendation | Lundkvist 2005 | Ramaekers 2013 | Sher 2018 | | Li 2020 | | |  |  |
|  | **Title and abstract** |  |  |  |  | |  | | |  |  |
| 1 | Title | Identify the study as an economic evaluation or use more specific terms such as “cost-effectiveness analysis”, and describe the interventions compared. | CRT not indicated | Not indicated | Yes | | IMRT not indicated | | |  |  |
| 2 | Abstract | Provide a structured summary of objectives, perspective, setting, methods (including study design and inputs), results (including base case and uncertainty analyses), and conclusions. | Perspective, setting, and model inputs not indicated | Perspective, setting and inputs not provided | Model inputs and conclusion not provided | | Perspective, model inputs and conclusion not provided | | |  |  |
|  | **Introduction** |  |  |  |  | |  | | |  |  |
| 3 | Background and objectives | Provide an explicit statement of the broader context for the study. | Yes | Yes | Yes | | Yes | | |  |  |
|  |  | Present the study question and its relevance for health policy or practice decisions. | Yes | Yes | Yes | | Yes | | |  |  |
|  | **Methods** |  |  |  |  | |  | | |  |  |
| 4 | Target population and subgroups | Describe characteristics of the base case population and subgroups analyzed, including why they were chosen. | Yes | Reason not explained | Reasons not explained | | Yes | | |  |  |
| 5 | Setting and location | State relevant aspects of the system(s) in which the decision(s) need(s) to be made. | Yes | Yes | Yes | | Yes | | |  |  |
| 6 | Study perspective | Describe the perspective of the study and relate this to the costs being evaluated. | Yes | Yes | Yes | | Yes | | |  |  |
| 7 | Comparators | Describe the interventions or strategies being compared and state why they were chosen. | Did not justify CRT as comparator until later in discussion | Yes | Yes | | Yes | | |  |  |
| 8 | Time horizon | State the time horizon(s) over which costs and consequences are being evaluated and say why appropriate. | Reasons not provided | Reasons not provided | Reasons not provided | | 2020 Chinese life expectancy | | |  |  |
| 9 | Discount rate | Report the choice of discount rate(s) used for costs and outcomes and say why appropriate. | Reasons not provided | Yes | Reasons not provided | | Yes | | |  |  |
| 10 | Choice of health outcomes | Describe what outcomes were used as the measure(s) of benefit in the evaluation and their relevance for the type of analysis performed. | Yes | Disease- and toxicity free life years (DTFLYs) were also included | Yes | | Yes | | |  |  |
| 11a | Measurement of effectiveness | Single study-based estimates: Describe fully the design features of the single effectiveness study and why the single study was a sufficient source of clinical effectiveness data. | N/A | The use of NTCP models for toxicity probabilities was well justified. A meta-analysis was used for transition probabilities. Cancer-related mortalities from loco-regional recurrence and distant metastases were derived from single studies and expert opinion with no justification | N/A | | N/A | | |  |  |
| 11b |  | Synthesis-based estimates: Describe fully the methods used for identification of included studies and synthesis of clinical effectiveness data. | Multiple sources were cited for mortality risk reduction, but no data synthesis reported | N/A | Multiple sources were cited for model inputs, but no data synthesis reported | | Multiple sources were cited for model inputs, but no data synthesis reported | | |  |  |
| 12 | Measurement and valuation of preference based outcomes | If applicable, describe the population and methods used to elicit preferences for outcomes. | Multiple sources were cited utility scores, but no data synthesis reported | Utility scores were derived from a cross-sectional study using the EQ-5D questionnaire in Dutch HNC patients | Utilities and disutilities were identified from 2 different cross-sectional studies | | Utilities were identified from 1 cross-sectional survey | | |  |  |
| 13a | Estimating resources and costs | Single study-based economic evaluation: Describe approaches used to estimate resource use associated with the alternative interventions. Describe primary or secondary research methods for valuing each resource item in terms of its unit cost. Describe any adjustments made to approximate to opportunity costs. | N/A | N/A | N/A | | N/A | | |  |  |
| 13b |  | Model-based economic evaluation: Describe approaches and data sources used to estimate resource use associated with model health states. Describe primary or secondary research methods for valuing each resource item in terms of its unit cost. Describe any adjustments made to approximate to opportunity costs. | Provided overview of the costing studies cited, and methods to derive unit costs | Activity-based costing was used with resource use and unit prices derived based on guidelines, a cross-sectional survey or expert opinion | CPT codes were used to estimate treatment costs from Medicare perspective. | | Estimates provided by a proton center and a cancer center | | |  |  |
|  |  |  |  |  | Breakdown costs of building and financing proton center were presented but no source indicated | |  |  |  |  |  |
| 14 | Currency, price date, and conversion | Report the dates of the estimated resource quantities and unit costs. Describe methods for adjusting estimated unit costs to the year of reported costs if necessary. Describe methods for converting costs into a common currency base and the exchange rate. | Yes | Methods of conversion not specified | Yes | | Yes | | |  |  |
| 15 | Choice of model | Describe and give reasons for the specific type of decision-analytical model used. Providing a figure to show model structure is strongly recommended. | No reason was provided | Yes | No reason was provided | | Yes | | |  |  |
|  |  |  | A figure of model structure was presented |  | A figure of model structure was presented | | A figure of model structure was presented | | |  |  |
| 16 | Assumptions | Describe all structural or other assumptions underpinning the decision-analytical model. | Yes | Yes | Yes | | Yes | | |  |  |
| 17 | Analytical methods | Describe all analytical methods supporting the evaluation. This could include methods for dealing with skewed, missing, or censored data; extrapolation methods; methods for pooling data; approaches to validate or make adjustments (such as half cycle corrections) to a model; and methods for handling population heterogeneity and uncertainty. | Described methods used to derive investment cost, operation cost, transportation and accommodation cost | Half-cycle correction applied for QALYs and costs. | Breakdown costs of treatment, building and financing proton center were presented in appendixes | | None | | |  |  |
|  | **Results** |  |  |  |  | |  | | |  |  |
| 18 | Study parameters | Report the values, ranges, references, and, if used, probability distributions for all parameters. Report reasons or sources for distributions used to represent uncertainty where appropriate. Providing a table to show the input values is strongly recommended. | A table of input values was provided However, listed costs didn’t match the texts | List of parameters provided in appendixes. Reasons or sources for distributions not indicated | Table of parameters provided. Reasons or sources for distributions not indicated | | Table of parameters provided. Reasons or sources for distributions and ranges of parameters not indicated | | |  |  |
| 19 | Incremental costs and outcomes | For each intervention, report mean values for the main categories of estimated costs and outcomes of interest, as well as mean differences between the comparator groups. If applicable, report incremental cost-effectiveness ratios. | Yes | A breakdown of costs not provided | Yes | | Only incremental costs and outcomes, incremental cost-effectiveness ratios | | |  |  |
| 20a | Characterizing uncertainty | Single study-based economic evaluation: Describe the effects of sampling uncertainty for the estimated incremental cost and incremental effectiveness parameters, together with the impact of methodological assumptions (such as discount rate, study perspective). | N/A | N/A | N/A | | N/A | | |  |  |
| 20b |  | Model-based economic evaluation: Describe the effects on the results of uncertainty for all input parameters, and uncertainty related to the structure of the model and assumptions. | Results from sensitivity scenarios presented in a table. Uncertainty related to the structure of the model and assumptions not tested | Unequal disease progression and survival for IMPT and IMRT were tested in alternative scenario. PSA and VOI also performed | One-way sensitivity analyses, PSA and VOI were performed and reported. Uncertainty related to the structure of the model and assumptions not tested | | One-way sensitivity analyses, PSA and stratefied analyses were performed and reported. Uncertainty related to the structure of the model and assumptions not tested | | |  |  |
| 21 | Characterizing heterogeneity | If applicable, report differences in costs, outcomes, or cost-effectiveness that can be explained by variations between subgroups of patients with different baseline characteristics or other observed variability in effects that are not reducible by more information. | N/A | IMPT if efficient strategy finds IMPT being cost-effective when treating only patients with higher expected benefit | HPV-positive and -negative populations were analyzed separately | | Stratefied analysis by age | | |  |  |
|  | **Discussion** |  |  |  |  | |  | | |  |  |
| 22 | Study findings, limitations, generalizability, and current knowledge | Summaries key study findings and describe how they support the conclusions reached. Discuss limitations and the generalizability of the findings and how the findings fit with current knowledge. | Yes | Yes | Generalizability of the findings not discussed | | Generalizability of the findings not discussed. Failed to discuss previous studies such as Lundkvist 2005 and Ramaekers 2013 | | |  |  |
|  | **Other** |  |  |  |  | |  | | |  |  |
| 23 | Source of funding | Describe how the study was funded and the role of the funder in the identification, design, conduct, and reporting of the analysis. Describe other non-monetary sources of support. | Yes | Yes | Not reported | | Yes | | |  |  |
| 24 | Conflicts of interest | Describe any potential for conflict of interest of study contributors in accordance with journal policy. In the absence of a journal policy, we recommend authors comply with International Committee of Medical Journal Editors recommendations. | Yes | Yes | Yes | | Yes | | |  |  |

| Supplementary Table 2. Quality assessment of decision-analytic models |  |  |  |  |  |  |  | |  | |  | |  |
| --- | --- | --- | --- | --- | --- | --- | --- | --- | --- | --- | --- | --- | --- |
|  |  | Lundkvist 2005 | | | | | | Ramaekers 2013 | | Sher 2018 | | Li 2020 | |
|  |  | Response | Comments | Response | Comments | Response | Comments | | Response | | Comments | |  |
| Dimension of quality | Questions for critical appraisal |  |  |  |  |  |  | |  | |  | |  |
| S1 Statement of decision | Is there a clear statement of the decision problem? | Yes | Whether the medical benefits of proton therapy justify the higher costs. | Yes | Comparative effectiveness evidence is sparsely available for proton radiation therapy for evidence-based decision making under uncertainty | Yes | PBT may lead to an improved subacute side effect profile but with substantially higher cost, which makes the cost-effectiveness of PBT an important and pressing question | | Yes | | PBT related costs are not yet covered by Chinese public medical insurance due to limited medical resources. So CEA is needed in clinical decision making for the appropriate radiotherapy mode | |  |
| problem/objective |  |  |  |  |  |  |  |  |  |  |  |  |  |
|  | Is the objective of the evaluation and model specified and consistent with the stated decision problem? | Yes | An economic evaluation of proton radiation therapy. | Yes | To combine NTCP models and comparative planning data in a model-based economic evaluation to explore the (cost-) effectiveness of swallowing-sparing IMPT (scanned) compared with swallowing sparing IMRT for HNC patients. | Yes | This analysis aims to determine the cost-effectiveness of PBT under a variety of toxicity assumptions and to assess whether its potential benefits may be sufficiently valuable to warrant a future randomized trial. | | Yes | | To evaluate the costeffectiveness of PBT with Chinese settings and to facilitate the decision making for paranasal sinus and nasal cavity cancer treatment | |  |
|  | Is the primary decision-maker specified? | No | Primary decision maker implied but not specified. | No | Not specified. | No | Not specified. | | No | | Not specified. | |  |
| S2 Statement of | Is the perspective of the model stated clearly? | Yes | Societal perspective | Yes | Dutch healthcare perspective | Yes | Payer (Medicare) and societal perspective | | Yes | | Chinese healthcare perspective | |  |
| scope/perspective |  |  |  |  |  |  |  |  |  |  |  |  |  |
|  | Are the model inputs consistent with the stated perspective? | No | From a societal perspective, the study included direct non-medical cost. However, indirect cost was not included. | Yes | Only direct medical costs were included | No | From a societal perspective, the study included direct non-medical cost. However, indirect cost was not included. | | Yes | | Only direct medical costs were included | |  |
|  | Has the scope of the model been stated and justified? | Yes | The model was for patients with head and neck cancers. Therefore some assumptions of cost and utility value were cancer specific. | Yes | The model was for locally advanced (stage III-IV) HNC patients (oral cavity, laryngeal, and pharyngeal cancer), aged on average 61 years at start of radiation therapy and pretreatment | Yes | The model was for locally advanced oropharyngeal carcinoma | | Yes | | The model was for paranasal sinus and nasal cavity cancers | |  |
|  |  |  |  |  | Radiation Therapy Oncology Group (RTOG) grade <2 dysphagia |  |  |  |  |  |  |  |  |
|  |  |  |  |  | and xerostomia. |  |  |  |  |  |  |  |  |
|  | Are the outcomes of the model consistent with the perspective, scope and overall objective of the model? | Yes | Common outcomes of cost, QALYs, and ICER | Yes | Expected mean costs, occurrence of toxicity, disease- and toxicity-free life years (DTFLYs) and QALYs | Yes | Cost, QALY and ICER | | Yes | | Cost, QALY and ICER | |  |
| S3 Rationale for structure | Has the evidence regarding the model structure been described? | No | Not specified. | Yes | Reflect of course of disease | Yes | Model structure was based on the clinical history of a 65-year-old patient with T2, N2, OPC receiving 35 fractions of bilateral chemoradiotherapy with bolus cisplatin. | | Yes | | Model structure was based on an old published efficacy analysis of 231 cases | |  |
|  | Is the structure of the model consistent with a coherent theory of the health condition under evaluation? | No | Over simplified model structure only consider health state with chronic adverse event besides healthy and death. Did not take into account the progression of disease. | No | Toxicities other than xerostomia and dysphagia not included | Yes |  | | Yes | | With simple model structure, authors claimed that clinical outcomes other than tumor control had no difference between IMPT and IMRT in theory and practice | |  |
|  | Have any competing theories regarding model structure been considered? | No | Not specified. | No | Not specified. | No | Not specified. | | No | | Not specified. | |  |
|  | Are the sources of data used to develop the structure of the model specified? | No | No source or justification provided. | No | No source provided. | Yes |  | | Yes | |  | |  |
|  | Are the causal relationships described by the model structure justified appropriately? | No | Not justified | No | Not justified | No | The reason of no progression from LRR to distant metastasis was not specified. | | Yes | | The advantage of IMPT compared with IMRT mainly lies in the improvement of tumor control due to the anatomical location and a relatively low radiosensitivity of paranasal sinus and nasal cavity cancer. A recent systematic review and meta-analysis found no significant reduction of acute and late toxicities. | |  |
| S4 Structural assumptions | Are the structural assumptions transparent and justified? | No | Assumptions presented but not justified. | Yes | Acute toxicity is reversible during the first 6 months after radiation therapy. Chronic toxicity is irreversible. No transition from locoregional recurrence to death via distant metastasis | No | No structural assumption reported | | Yes | | Other clinical outcomes such as toxicities were assumed to be identical between IMPT and IMRT | |  |
|  | Are the structural assumptions reasonable given the overall objective, perspective and scope of the model? | Yes | Reasonable given data availability. | Yes | Assumptions justified | N/A |  | | Yes | |  | |  |
| S5: Strategies/comparators | Is there a clear definition of the options under evaluation? | Yes | PBT vs. CRT | Yes | IMPT vs IMRT vs. IMPT if efficient | Yes | PBT vs. IMRT | | Yes | | IMPT vs. IMRT | |  |
|  | Have all feasible and practical options been evaluated? | No | IMRT not included. | Yes | IMRT was considered current practice | Yes | IMRT was considered current practice | | Yes | | IMRT was considered current practice | |  |
|  | Is there justification for the exclusion of feasible options? | Yes | Long-term data only available for CRT. | N/A |  | Yes |  | | No | |  | |  |
|  |  |  |  |  |  |  |  | |  | |  | |  |
|  |  |  |  |  |  |  |  | |  | |  | |  |
| S6: Model type | Is the chosen model type appropriate given the decision problem and specified causal relationships within the model? | Yes | A Markov cohort model was appropriate. | Yes | A Markov cohort model was appropriate. | Yes | A Markov cohort model was appropriate. | | Yes | | A Markov cohort model was appropriate. | |  |
| S7: Time horizon | Is the time horizon of the model sufficient to reflect all important differences between options? | Yes |  | Yes |  | Yes |  | | Yes | |  | |  |
|  | Is the time horizon of the model, and the duration of treatment and treatment effect described and justified? | No | Unclear how long the treatment effect lasted. | No | Duration treatment effect unclear. One-time treatment effect on toxicity reduction was implied | No | Unclear how long the treatment effect lasted. | | No | | Treatment effect was one-time | |  |
|  | Has a lifetime horizon been used? If not, has a shorter time horizon been justified? | Yes |  | Yes |  | Yes |  | | Yes | |  | |  |
| S8: Disease states/pathways | Do the disease states (state transition model) or the pathways (decision tree model) reflect the underlying biological process of the disease in question and the impact of interventions? | No | Cancer progression was not considered in the model. | No | Toxicities other than xerostomia and dysphagia not included | No | Unclear why no progression from LRR to distant metastasis. Other toxicities not included | | No | | Simple model with states of no cancer, live with cancer, and deadth | |  |
| S9: Cycle length | Is the cycle length defined and justified in terms of the natural history of disease? | N/A |  | Yes | 6-month cycle was used in the first year to account for the reversibility of acute toxicity | No | No justification for 1 month cycle length | | No | | No justification for yearly cycle | |  |
| **Data (D)** |  |  |  |  |  |  |  | |  | |  | |  |
| D1: Data identification | Are the data identification methods transparent and appropriate given the objectives of the model? | No | Not specified. | Yes | The process to derive probabilities of toxicity using NTCP models were explained | No | Breakdown costs of building and financing proton center were presented but no source indicated | | No | | Not specified. | |  |
|  | Where choices have been made between data sources, are these justified appropriately? | No | Not specified. | No | Not specified. | No | Not specified. | | No | | Not specified. | |  |
|  | Has particular attention been paid to identifying data for the important parameters in the model? | Yes | The calculations of proton therapy investment and operation costs were presented. However, the estimation of mortality risk reduction lacked details. | Yes | NTCP models were combined with comparative planning studies to calculate probabilities of toxicity | Yes | Relative risks of toxicities treated with PBT were important. However, assumptions were used due to the lack of evidence | | Yes | | Probabilities of IMPT and IMRT eradicating cancer were deemed important and identified from systematic reviews of observational studies | |  |
|  | Has the process of selecting key parameters been justified and systematic methods used to identify the most appropriate data? | No | Not specified. | No | Not specified. | No | Not specified. | | No | | Not specified. | |  |
|  | Has the quality of the data been assessed appropriately? | No | Not specified. | No | Not specified. | No | Not specified. | | No | | Not specified. | |  |
|  | Where expert opinion has been used, are the methods described and justified? | N/A |  | No | Not specified. | No | Not specified. | | Yes | | Cost data were estimates can proton center and cancer certers | |  |
| D2: Pre-model data analysis | Are the pre-model data analysis methodology based on justifiable statistical and epidemiological techniques? | N/A |  | Yes | NTCP models were validated with photons | N/A |  | | N/A | |  | |  |
| D2a: baseline data | Is the choice of baseline data described and justified? | Yes/No | Cancer mortality rate estimated from the Swedish cancer registry, but no detail provided | No | A planning study with small sample size was used | Yes | Transition probabilities were identified from RTOG trials | | Yes | | Transition probabilities were identified from systematic reviews of observational studies | |  |
|  | Are transition probabilities calculated appropriately? | No | Transition probabilities were not presented except for cancer mortality rate. | N/A | Calculations were not presented | N/A | Calculations were not presented | | N/A | | Calculations were not presented | |  |
|  | Has a half cycle correction been applied to both cost and outcome? | No | Not specified/not applied. | Yes |  | No |  | | No | |  | |  |
|  | If not, has this omission been justified? | No |  | N/A |  | No |  | | No | |  | |  |
| D2b: treatment effects | If relative treatment effects have been derived from trial data, have they been synthesized using appropriate techniques? | No | Not specified/not applied | No | Not specified | No | Not specified | | No | | Not applied | |  |
|  | Have the methods and assumptions used to extrapolate short-term results to final outcomes been documented and justified? Have alternative assumptions been explored through sensitivity analysis? | No | No extrapolation applied. No alternative assumptions tested. | Yes | Alternative assumption tested in sensitivity analysis | No | No extrapolation specified | | No | | No extrapolation | |  |
|  | Have assumptions regarding the continuing effect of treatment once treatment is complete been documented and justified? Have alternative assumptions been explored through sensitivity analysis? | No | Continuing effect of mortality reduction was not justified. No alternative assumptions tested. | No | Continuing effect assumed but not justified. No alternative assumptions tested. | No | Continuing effect implied but not justified. No alternative assumptions tested. | | No | | No continuing effect | |  |
|  |  |  |  |  |  |  |  | |  | |  | |  |
| D2c: quality-of-life weights (utilities) | Are the utilities incorporated into the model appropriate? | No | Only one utility score was provided. Unclear whether utilities vary across health states or adverse events. | Yes | EQ-5D administered in Dutch HNC patients in a cross-sectional study | Yes | Direct utilities and disutilities extracted from cross-sectional studies | | No | | Utilities came from a previous study of patients with squamous cell carcinoma of the upper aerodigestive tract using Time Trade-Off method | |  |
|  | Is the source for the utility weights referenced? | Yes | Utility weight used referenced. | Yes | Utility weight used referenced. | Yes | Utility weight used referenced. | | Yes | | Utility weight used referenced. | |  |
|  | Are the methods of derivation for the utility weights justified? | No | The derivation for the utility weights was not specified. | N/A | No utility derivation in the study | N/A | No utility derivation in the study | | N/A | | No utility derivation in the study | |  |
| D3: Data incorporation | Have all data incorporated into the model been described and referenced in sufficient detail? | No | The calculations of proton therapy investment and operation costs were presented. However, the estimation of mortality risk reduction lacked details. | Yes | List of data with references presented in appendixes | No | Breakdown costs of building and financing proton center were presented but no source indicated | | Yes | | Table of data with references provided | |  |
|  | Has the use of mutually inconsistent data been justified (i.e. are assumptions and choices appropriate)? | N/A | Unclear if mutually inconsistent data was used. | N/A | Unclear if mutually inconsistent data was used. | N/A | Unclear if mutually inconsistent data was used. | | N/A | | Unclear if mutually inconsistent data was used. | |  |
|  | Is the process of data incorporation transparent? | No | The estimation of mortality risk reduction was unclear. | Yes | The process to derive probabilities of toxicity using NTCP models were explained | Yes | The use of point estimates and distributions was presented | | No | | 90% CI for model parameters were calculated from probabilistic sensitivity analysis with 50,000 iteration trials, instead of CI from the references | |  |
|  | If data have been incorporated as distributions, has the choice of distribution for each parameter been described and justified? | N/A |  | No | The choice of distribution not justified | No | The choice of distribution not justified | | No | | The choice of distribution not justified | |  |
|  | If data have been incorporated as distributions, is it clear that second order uncertainty is reflected? | N/A | No PSA conducted | Yes | Monte Carlo simulation with 20,000 iterations | Yes | Monte Carlo simulation with 50,000 iterations | | Yes | | Monte Carlo simulation with 50,000 iterations | |  |
| D4: Assessment of uncertainty | Have the four principal types of uncertainty been addressed? | No | Only parameter uncertainty was tested in scenario analyses. | No | Methodological uncertainty not addressed | No | Methodological and structural uncertainty not addressed | | No | | Methodological and structural uncertainty not addressed | |  |
|  | If not, has the omission of particular forms of uncertainty been justified? | No |  | No |  | No |  | | No | |  | |  |
| D4a: methodological | Have methodological uncertainties been addressed by running alternative versions of the model with different methodological assumptions? | No |  | No |  | No |  | | No | |  | |  |
| D4b: structural | Is there evidence that structural uncertainties have been addressed via sensitivity analysis? | No |  | Yes | Unequal disease progression and survival for IMPT and IMRT were tested in alternative scenario | No |  | | No | |  | |  |
| D4c: heterogeneity | Has heterogeneity been dealt with by running the model separately for different sub-groups? | No |  | Yes | IMPT if efficient strategy finds IMPT being cost-effective when treating only patients with higher expected benefit | Yes | HPV-positive and -negative populations were analyzed separately | | Yes | | Stratefied analysis by age categoriy | |  |
| D4d: parameter | Are the methods of assessment of parameter uncertainty appropriate? | Yes |  | Yes |  | Yes |  | | No | | 90% CI for model parameters were calculated from probabilistic sensitivity analysis with 50,000 iteration trials, instead of CI from the references | |  |
|  | Has probabilistic sensitivity analysis been done, if not has this been justified? | N/A |  | Yes |  | Yes |  | | Yes | |  | |  |
|  | If data are incorporated as point estimates, are the ranges used for sensitivity analysis stated clearly and justified? | Yes/No | +25%, +/-50%, -75% and -90% of hazard rate were tested but unjustified. Proton facility lifetime was also varied in sensitivity scenario analyses but unjustified. | Yes | Standard errors were used | Yes/No | Ranges presented but not justified | | Yes | | 90% CI for model parameters were calculated from probabilistic sensitivity analysis with 50,000 iteration trials | |  |
| **Consistency (C)** |  |  |  |  |  |  |  | |  | |  | |  |
| C1: Internal consistency | Is there evidence that the mathematical logic of the model has been tested thoroughly before use? | No | No consistency checks are reported. | No | No consistency checks are reported. | No | No consistency checks are reported. | | No | | No consistency checks are reported. | |  |
| C2: External consistency | Are the conclusions valid given the data presented? | Yes |  | Yes |  | Yes |  | | Yes | |  | |  |
|  | Are any counterintuitive results from the model explained and justified? | N/A |  | N/A |  | N/A |  | | N/A | |  | |  |
|  | If the model has been calibrated against independent data, have any differences been explained and justified? | No | No calibration conducted. | No | No calibration conducted. | Yes | The model was calibrated to the locoregional control and distant metastasis results from RTOG 0129. The authors suggested that (1) the cumulative incidence of distant metastasis was lower in the HPV-negative population because of the competing risk of locoregional failure; and (2) the overestimation of survival in the HPV-negative population may be due to competing risks of death in this population that are not included in standard life tables | | No | | No calibration conducted. | |  |
|  | Have the results of the model been compared with those of previous models and any differences in results explained? | No | No comparison performed. | Yes | Compared with Lundkvist 2005 | Yes | Compared with Lundkvist 2005 and Ramaekers 2013 | | Yes | | Compared with Sher 2018 | |  |

| Supplementary Table 3. Hierarchy scale for data sources in economic evaluations | |  |  |  |  |
| --- | --- | --- | --- | --- | --- |
| **Clinical effect sizes/adverse events and complications** | | **Lundkvist 2005** | **Ramaekers 2013** | **Sher 2018** | **Li 2020** |
| **1+** | Meta-analysis of RCTs with direct comparison between comparator therapies, measuring final outcomes |  |  |  |  |
| **1** | Single RCT with direct comparison between comparator therapies, measuring final outcomes |  |  |  |  |
| **2+** | Meta-analysis of RCTs with direct comparison between comparator therapies, measuring surrogate outcomes |  |  |  |  |
|  | Meta-analysis of placebo-controlled RCTs with similar trial populations, measuring the final outcomes for each individual therapy |  |  |  |  |
| **2** | Single RCT with direct comparison between comparator therapies, measuring the surrogate outcomes |  |  |  |  |
|  | Single placebo-controlled RCTs with similar trial populations, measuring the final outcomes for each individual therapy |  |  |  |  |
| **3+** | Meta-analysis of placebo-controlled RCTs with similar trial populations, measuring the surrogate outcomes |  |  |  |  |
| **3** | Single placebo-controlled RCTs with similar trial populations, measuring the surrogate outcomes for each individual therapy |  |  | X |  |
| **4** | Case control or cohort studies | X | X | X | X |
| **5** | Non-analytic studies (e.g. case reports, case series) |  | X |  |  |
| **6** | Expert opinion | X |  |  |  |
| **9** | Not clearly stated |  |  |  |  |
| **Baseline clinical data (if applicable)** | | N/A |  |  |  |
| **1** | Case series or analysis of reliable administrative databases specifically conducted for the study covering patients solely from the jurisdiction of interest |  |  |  |  |
| **2** | Recent case series or analysis of reliable administrative databases covering patients solely from the jurisdiction of interest |  | X | X |  |
| **3** | Recent case series or analysis of reliable administrative databases covering patients solely from another jurisdiction |  |  |  |  |
| **4** | Old case series or analysis of reliable administrative databases. Estimates from RCTs. |  |  |  | X |
| **5** | Estimates from previously published economic analyses: unsourced |  |  |  |  |
| **6** | Expert opinion |  |  |  |  |
| **9** | Not clearly stated |  |  |  |  |
| **Resource use** | |  |  |  |  |
| **1** | Prospective data collection or analysis of reliable administrative data for specific study |  |  |  |  |
| **2** | Recently published results of prospective data collection or recent analysis of reliable administrative data: same jurisdiction |  |  |  |  |
| **3** | Unsourced data from previous economic evaluations: same jurisdiction |  |  |  |  |
| **4** | Recently published results of prospective data collection or recent analysis of reliable administrative data: different jurisdiction |  |  |  |  |
| **5** | Data source not known: different jurisdiction |  |  |  |  |
| **6** | Expert opinion | X | X | X | X |
| **9** | Not clearly stated |  |  |  |  |
| **Costs** | |  |  |  |  |
| **1** | Cost calculations based on reliable databases or data sources conducted for specific study: same jurisdiction |  | X | X |  |
| **2** | Recently published cost calculations based on reliable databases or data course: same jurisdiction |  |  |  |  |
| **3** | Data source not known: same jurisdiction |  |  |  | X |
| **4** | Using charge (price) rather than cost when societal perspective was adopted |  |  |  |  |
| **5** | Recently published cost calculations based on reliable databases or data sources: different jurisdiction | X | X |  |  |
| **6** | Data source not known: different jurisdiction |  |  |  |  |
| **9** | Not clearly stated |  |  |  |  |
| **Utilities (if applicable)** | |  |  |  |  |
| **1** | Direct utility assessment for the specific study from a sample either: |  |  |  |  |
|  | (a) of the general population, or |  |  |  |  |
|  | (b) with knowledge of the disease(s) of interest, or |  |  |  |  |
|  | (c) of patients with the disease(s) of interest |  |  |  |  |
|  | Indirect utility assessment from specific study from patient sample with disease(s) of interest, using a tool validated for the patient population |  |  |  |  |
| **2** | Indirect utility assessment from a patient sample with disease(s) of interest, using a tool not validated for the patient population |  |  |  |  |
| **3** | Direct utility assessment from a previous study from a sample either: |  |  |  |  |
|  | (a) of the general population, or |  |  |  |  |
|  | (b) with knowledge of the disease(s) of interest, or |  |  |  |  |
|  | (c) of patients with the disease(s) of interest |  |  | X | X |
|  | Indirect utility assessment from previous study from patient sample with disease(s) of interest, using a tool validated for the patient population |  | X |  |  |
| **4** | Data source not known: method of elicitation unknown | X |  |  |  |
| **5** | Patient preference values obtained from a visual analogue scale |  |  |  |  |
| **6** | Delphi panels, expert opinion |  |  |  |  |
| **9** | Not clearly stated |  |  |  |  |
